# Reduced sensitivity to both positive and negative reinforcement in mice over‐expressing the 5‐hydroxytryptamine transporter

**DOI:** 10.1111/ejn.12744

**Published:** 2014-10-04

**Authors:** Samantha J. Line, Chris Barkus, Nancy Rawlings, Katie Jennings, Stephen McHugh, Trevor Sharp, David M. Bannerman

**Affiliations:** ^1^ Department of Experimental PsychologyThe University of OxfordSouth Parks RoadOxfordOX1 3UDUK; ^2^ Department of Physiology, Anatomy and GeneticsThe University of OxfordOxfordUK; ^3^ Department of PharmacologyThe University of OxfordOxfordUK

**Keywords:** decision‐making, fear conditioning, serotonin, spatial memory

## Abstract

The 5‐hydroxytryptamine (5‐HT) transporter (5‐HTT) is believed to play a key role in both normal and pathological psychological states. Much previous data suggest that the *s* allele of the polymorphic regulatory region of the 5‐HTT gene promoter is associated with reduced 5‐HTT expression and vulnerability to psychiatric disorders, including anxiety and depression. In comparison, the *l* allele, which increases 5‐HTT expression, is generally considered protective. However, recent data link this allele to both abnormal 5‐HT signalling and psychopathic traits. Here, we studied the processing of aversive and rewarding cues in transgenic mice that over‐express the 5‐HTT (5‐HTTOE mice). Compared with wild‐type mice, 5‐HTTOE mice froze less in response to both a tone that had previously been paired with footshock, and the conditioning context. In addition, on a decision‐making T‐maze task, 5‐HTTOE mice displayed reduced preference for a larger, delayed reward and increased preference for a smaller, immediate reward, suggesting increased impulsiveness compared with wild‐type mice. However, further inspection of the data revealed that 5‐HTTOE mice displayed a relative insensitivity to reward magnitude, irrespective of delay. In contrast, 5‐HTTOE mice appeared normal on tests of spatial working and reference memory, which required an absolute choice between options associated with either reward or no reward. Overall, the present findings suggest that 5‐HTT over‐expression results in a reduced sensitivity to both positive and negative reinforcers. Thus, these data show that increased 5‐HTT expression has some maladaptive effects, supporting recent suggestions that *l* allele homozygosity may be a potential risk factor for disabling psychiatric traits.

## Introduction

The 5‐hydroxytryptamine (5‐HT; serotonin) transporter (5‐HTT; SERT) gene (SLC6A4) exhibits several common polymorphisms that may modulate expression, activity and/or regulation of the transporter protein (Murphy & Moya, 2011). One important 5‐HTT polymorphism is an insertion/deletion of a repetitive sequence located in the proximal 5′ regulatory region of the gene promoter (5‐HTT gene‐linked polymorphic region; 5‐HTTLPR), which gives rise to long (*l*) and short (*s*) alleles, respectively. Studies examining the functional effect of this polymorphism report that the *l* allele displays greater 5‐HTT expression and/or activity than the *s* allele. Moreover, it has been suggested that the polymorphism influences brain activity, personality, susceptibility to psychiatric illness and response to psychotropic drugs (Lesch *et al*., 1996; Caspi *et al*., 2003; Lee *et al*., 2004; Hariri *et al*., 2005; Homberg & Lesch, 2011; Murphy & Moya, 2011), although these findings are not without controversy (Risch *et al*., 2009; Uher & McGuffin, 2010).

Compared with *l* allele carriers, *s* allele homozygosity has been associated with increased anxiety‐related personality traits (Sen *et al*., 2004), increased processing of fearful stimuli (Hariri *et al*., 2005; Osinsky *et al*., 2008) and enhanced fear conditioning (Garpenstrand *et al*., 2001). Indeed, the *s* allele is generally considered a risk factor for a number of psychiatric disorders, including depression and anxiety (Murphy & Moya, 2011). Consistent with this, 5‐HTT knockout (KO) mice display relevant phenotypes, including increased anxiety, impaired extinction recall of fear memories and increased sensitivity to stress (Holmes *et al*., 2003; Adamec *et al*., 2006; Wellman *et al*., 2007; Line *et al*., 2011).

In contrast, the *l* allele has been widely considered protective. However, evidence is accumulating that *l* allele homozygosity may also have maladaptive effects, and could represent a potential risk factor for the development of psychiatric conditions (Glenn, 2011). 5‐HTT over‐expressing (5‐HTTOE) mice have been engineered (Jennings *et al*., 2006) and represent an important experimental tool for investigating behavioural effects of changes in 5‐HTT expression. These mice exhibit increased 5‐HTT expression within the physiological range. Previous studies found a decrease in unconditioned anxiety in the 5‐HTTOE mice (Jennings *et al*., 2006; Line *et al*., 2011), thereby complementing the increased anxiety in 5‐HTT KO mice. However, recent *in vitro* neurochemical analysis revealed that not only loss, but also gain, of 5‐HTT expression impaired phasic 5‐HT transmission, suggesting an inability to appropriately relay 5‐HT‐mediated information (Jennings *et al*., 2010).

It has long been suggested that altered mood may reflect differences in associative learning processes, and the sensitivity to positive and negative reinforcers (Beck, 1976). Here we assessed 5‐HTTOE mice on associative learning tasks, using both appetitively and aversively motivated conditioning procedures. We recently reported impaired fear conditioning in male 5‐HTTOE mice (Barkus *et al*., 2014). Here we repeat this fear conditioning study in female mice. In addition, we examined the performance of female 5‐HTTOE mice on a decision‐making task that assessed impulsive choice, and requires animals to integrate information about reward and delay to reinforcement.

## Materials and methods

### Animals

Experiments were conducted in accordance with the United Kingdom Animals (Scientific Procedures) Act of 1986, and following review by the Local Ethical Review Committee for the Departments of Experimental Psychology and Physiology, Anatomy and Genetics at Oxford University. 5‐HTTOE mice and wild‐type littermates were generated by mating wild‐type CBA × C57BL/6J females (obtained from Harlan, UK) with male heterozygous mice, as described previously (Jennings *et al*., 2006). Breeding took place at the University of Oxford. Female animals were used throughout. Mice were group housed and provided with sawdust bedding, nesting material and cardboard enrichment, and maintained on a 12 h light/dark cycle (lights off 19:00–07:00 h) in a temperature‐controlled environment (21 ± 1 °C). Separate cohorts of female animals were used for: (i) fear conditioning; (ii) cost/benefit decision‐making; and (iii) spatial learning experiments.

### Behavioural protocols

#### Fear conditioning

Fear conditioning took place in an operant chamber (17 cm long × 11.5 cm wide × 20 cm high) located in a sound‐insulated box. The walls and lid of the chamber (illuminated by a ceiling‐mounted light) were composed of clear Perspex, whilst the floor of the chamber consisted of 19 stainless‐steel bars approximately 8.5 mm apart, through which scrambled shocks were delivered (0.3 mA, 0.5 s duration; San Diego Instruments shock generator). During training and context testing, a plastic cube scented with artificial ‘apple pie’ odour (Dale Air, Rochdale, UK) was placed next to the conditioning chamber inside the sound‐insulated box. Between each trial all faeces/urine were removed and the boxes were cleaned with 70% alcohol and allowed to dry.

Black‐and‐white video images of the mice were captured by a wide‐angle video camera attached to the ceiling of the chamber, and relayed to a computer via a Panasonic video recorder (NV‐SD400). Video data were analysed using a Videotrack (vNT4.0) automated tracking system (Viewpoint, Champagne Au Mont D'Or, France) with a low‐ and high‐activity threshold setting. ‘Freezing’ was defined as periods during which movement fell below the lower activity threshold. With this threshold, breathing movements did not register as activity (i.e. absence of freezing), but small purposeful movements (e.g. sniffing) were detected as activity. The lower threshold was carefully calibrated using test pilot 5‐HTTOE and wild‐type animals. Movements above the upper threshold registered as very rapid ‘burst’ activity, comprising quick, darting movements and attempted escape responses. Thresholds were validated by manual observation (off‐line) of the animals’ behaviour. For most analyses the amount of time spent freezing per 30‐s time bin was calculated. For measurement of the unconditioned response to the tone and the unconditioned burst activity response to the shock, time bins of 1 s were used.

The fear conditioning protocol comprised three experimental sessions. A training session (13 min) was performed on day 1, and two test sessions (‘cue’ and ‘context’ tests) were performed 24 h later. The training session began with a 6‐min acclimatisation period, during which white noise occurred. This was followed by a 30‐s auditory tone. On tone offset mice received a 0.5‐s footshock (0.3 mA). After a further 3 min of white noise, a second tone/shock pairing was delivered, followed by a further 3‐min period of white noise only.

Twenty‐four hours after the training session, mice underwent both a ‘cue’ and ‘context’ testing session, which were counterbalanced so that half of each genotype group received the cue test first, and half received the context test first. During cue testing, mice were exposed to the tone in a novel environmental context (a round plastic chamber with patterned walls, smooth floor and a distinctive ‘chicken’ odour; Dale Air). Mice experienced two 30‐s presentations of the tone (without footshock) during the 5‐min session. During context testing, mice were placed back in the Perspex operant chamber that had been used for training, and experienced white noise for 5 min. Activity and freezing levels were recorded throughout each session.

#### Delay cost/benefit decision‐making

The performance of 5‐HTTOE mice was also examined on a cost/benefit decision‐making task that assesses impulsive choice. Performance on this task is sensitive to reductions in 5‐HT availability (Denk *et al*., 2005; see also Bizot *et al*., 1999; Mobini *et al*., 2000), a neurochemical phenotype displayed by the 5‐HTTOE mice (Jennings *et al*., 2006, 2010). Mice were given a choice between one goal arm that contained an immediately available, low reward (LR arm), and a second goal arm that contained a delayed, high reward (HR arm).

##### Shaping

Animals first underwent food restriction and structured exposure to diluted sweetened condensed milk. Their weight was maintained at approximately 90% of free‐feeding weight throughout testing.

##### Apparatus

**Figure 1:**
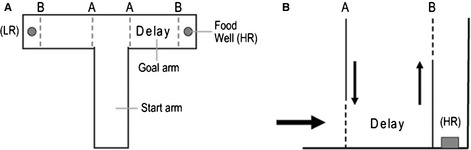
Apparatus for cost/benefit decision‐making experiment. (A) T‐maze seen from above. One goal arm always contained a low reward (LR) and one arm always contained a high reward (HR). Guillotine doors were located at the entrances of the goal arms (A) and in front of the food wells (B). (B) Lateral view of the goal arm to demonstrate movement of the doors for introduction of a delay.

Testing was performed using an enclosed T‐maze painted grey, consisting of a start arm and two identical goal arms (all 30 × 10 cm with 30‐cm‐high walls), with raised metal food wells (12 mm diameter) at the far end of each goal arm. Gates that could be lifted and lowered were present at the entrances to the goal arms and directly in front of the food wells (Fig. 1). For each animal, one arm was designated as the HR arm (containing 0.25 mL diluted, sweetened, condensed milk) and one arm was designated the LR arm (containing 0.05 mL diluted sweetened condensed milk). This assignment remained constant throughout testing for an individual animal. For half of the animals the HR was in the right arm, and for the other half it was associated with the left arm (this assignment was counterbalanced across genotypes).

##### Experimental design

The experiment was divided into five phases (50 choice trials per phase), with each phase consisting of five blocks of testing, and each block consisting of 10 trials (two consecutive days of testing with five choice trials/day). The reward allocations in the two goal arms remained the same throughout all phases. Phases differed in the length of the delay animals had to endure before receiving food rewards as follows:

1. Phase 1 – reward discrimination training; no delay in either arm;
2. Phase 2 – HR delayed by 5 s, LR immediate;
3. Phase 3 – HR delayed by 10 s, LR immediate;
4. Phase 4 – HR delayed by 10 s, LR delayed by 10 s;
5. Phase 5 – HR delayed by 10 s, LR immediate (as during Phase 3).

##### Reward discrimination training

At the start of the experiment mice were first given 12 forced trials (six to each arm), during which the door to one of the goal arms was closed. This ensured exposure to the reward magnitudes available in both arms. On subsequent days mice were given two forced trials (one to each arm), followed by five choice trials, during which the doors to both goal arms were open. During the choice trials animals were placed in the start arm and allowed to choose either goal arm. The numbers of HR arm choices made by the mice were recorded. All animals received 50 choice trials without any delay in either arm. By this stage of training, all mice were choosing the HR arm on the majority of trials. Nevertheless, a criterion of ≥ 80% HR choices during the final three blocks of Phase 1 was introduced. Animals that failed to meet this criterion were excluded from subsequent phases of the experiment.

##### Delay cost/benefit decision‐making

A delay to reinforcement was then introduced into the HR arm. As before, mice were given two forced trials each day (one to each arm), followed by five choice trials (during which the doors to both goal arms were open). Delay and reinforcement conditions on the forced trials were the same as during the subsequent choice trials on a given day. At the start of each choice trial the doors at the entrances to the goal arms were open (labelled A in Fig. 1), whilst those in front of the food wells were closed (labelled B). After entering a goal arm, door A (at the entrance of that arm) was closed. Depending on the phase of the task and the corresponding delay condition, door B would then either be opened immediately, or following a delay (during which the animal was contained in the goal arm). The opening of door B gave the animal access to the condensed milk reward.

Mice received 50 trials with a 5‐s delay to reinforcement in the HR arm (Phase 2). This delay was then increased to 10 s (Phase 3). In Phase 4, an equal delay to reinforcement of 10 s was introduced into both the HR and LR arms. Finally, during Phase 5, the conditions from Phase 3 were re‐introduced with a 10‐s delay to reinforcement in the HR arm and immediate access to the reward in the LR arm. As most mice were choosing the HR arm on the majority of trials at the end of Phase 4, and therefore would have been unaware of any change in conditions in the LR arm (i.e. that the delay to reinforcement was reduced from 10 to 0 s), 20 forced trials with the new conditions were given between Phases 4 and 5 (10 forced trials to the HR arm and 10 to the LR arm).

Finally, given the difference in the rate at which the two groups of mice acquired the differential reward discrimination task (Phase 1), this was subsequently repeated using a second cohort of experimentally naïve mice. All shaping and testing procedures were as described above.

#### Spatial memory

Spatial memory was assessed using a number of paradigms. Short‐term, spatial memory (spatial working memory) was assessed using a test of rewarded alternation (non‐matching to place) on an elevated T‐maze. Associative long‐term, spatial memory (spatial reference memory) was assessed using both an appetitive (elevated Y‐maze reference memory task) and an aversive paradigm (the open‐field Morris water‐maze task).

##### Rewarded alternation

Spatial working memory (non‐matching to place) was carried out using a grey, wooden T‐maze, elevated 1 m above the floor in a well‐lit room. The maze consisted of a start arm (30 × 10 cm) and two identical goal arms (30 × 10 cm), surrounded by a 10‐cm‐high wall. Metal food wells were located 3 cm from the end of each goal arm. Prominent extramaze cues were present throughout the testing room. Prior to the start of testing, mice were food restricted to 90% of their free‐feeding weight and familiarised with the T‐maze, and to the 50% sweetened condensed milk reward (diluted with water).

During rewarded alternation testing, mice underwent five trials per day. Each trial was divided into a sample run and a choice run. At the start of each trial, 0.1 mL of diluted, sweetened, condensed milk was placed in the food wells at the ends of each goal arm. Mice were placed at the distal end of the start arm and allowed to run to the end of the goal arm. During the sample run, mice were forced to turn either left or right by the presence of a wooden block, according to a pseudorandom sequence. During the choice run, mice were given a free choice of either goal arm. A trial was scored as ‘correct’ if the animal entered the previously unvisited arm (i.e. if the mouse alternated). A total of 40 trials was performed by each mouse.

##### Reference memory Y‐maze

Associative, long‐term spatial memory (spatial reference memory) was examined using an appetitively motivated Y‐maze task. The maze was elevated 80 cm above the floor and had 0.5‐cm‐high walls. This allowed mice to view extramaze cues around the testing room. A metal food well was located at the distal end of each arm. Each mouse was designated an allocentrically defined target arm where it would receive a 0.1 mL diluted, sweetened, condensed milk reward. The target arm for each mouse remained the same throughout testing, and target arms were counterbalanced with respect to genotype. The start arm for each trial was determined by a pseudorandom sequence, with equal numbers of starts from each of the two remaining arms in any one session, and no more than three consecutive starts from the same arm. The mouse was placed at the distal end of the start arm, and allowed to run freely until it reached the distal end of either its target (rewarded) arm or the non‐rewarded arm. A correct choice occurred when the mouse entered the target arm, and the percentage of correct choices per block of 10 trials was recorded. Animals received 60 trials in total. The maze was rotated by 120° randomly in either a clockwise or anticlockwise direction between trials, to prevent the mice from identifying the correct target arm by utilising olfactory, visual or tactile intramaze cues.

##### Morris water‐maze

Associative, long‐term spatial memory was further assessed using the aversively motivated, open‐field Morris water‐maze task. The maze consisted of a large circular tank (diameter 2.0 m, depth 0.6 m) containing water at a temperature of 21 ± 1 °C and a depth of 0.3 m. In order to escape from the water, the mouse had to locate a hidden escape platform (circular, diameter 21 cm, covered in wire mesh) submerged approximately 0.5 cm below the surface of the water, which remained in a fixed location on every trial. Milk (1 L) was added to the water in order to obscure the platform and allow efficient tracking of the swim paths. The maze was located in a laboratory containing various prominent visual extramaze cues. The swim paths taken by the animals were monitored by a video camera mounted in the ceiling above the pool. During training mice were given four trials per day. The platform was located in the centre of either the northwest, northeast, southwest or southeast quadrant of the pool, and the number of animals trained to each platform location was counterbalanced within each group. The mice were placed into the pool facing the side wall at one of eight start locations (N, S, E, W, NE, NW, SE, SW), in a pseudorandom order, and allowed to swim until they found the platform, or for a maximum of 90 s. Any mouse that failed to find the platform within the 90 s was guided to its location by the experimenter. The mice were then allowed to remain on the platform for 30 s before commencing the next trial. The inter‐trial interval was approximately 15 s.

The acquisition phase of the experiment involved 6 days of training. On day 7 (24 h after the 6th day of training), a ‘probe test’ was conducted during which the platform was removed from the pool and the mouse allowed to swim freely for 60 s. The percentage of time spent in each quadrant of the maze was recorded, as well as the number of crossings of the four potential platform positions (‘annulus crossings’).

### Neurochemical analysis

High‐performance liquid chromatography with electrochemical detection was used to measure brain tissue levels of 5‐HT and 5‐hydroxyindole‐3‐acetic acid in mice that had completed the delay cost/benefit decision‐making task. Mice were killed by cervical dislocation, and brains were rapidly removed, frozen (isopentane plus dry ice) and stored at −70 °C. The frontal cortex, hippocampus, striatum and midbrain were subsequently dissected and weighed in 1 mL ice‐cold 0.1 m perchloric acid. Tissue was homogenised (10 s; polytron kinetic homogeniser), centrifuged (21 382 ***g***, 15 min) and supernatants were stored on ice prior to analysis.

Analytes were separated on Microsorb C18 reverse‐phase columns (4.6 × 100–150 mm) and detected (glassy carbon working electrode set at +0.7 V vs. Ag/AgCl; LC‐4B electrochemical detector). The mobile phase consisted of 12.5% methanol, 0.13 m NaH_2_PO_4_, 0.85 mm EDTA and 0.01 mm sodium octane sulphonate at pH 3.55 (1 mL/min flow rate). After analysis, metabolite concentrations in each sample were converted into pmol/mg wet tissue.

### Statistical analysis

All data were analysed with repeated‐measures analysis of variance (anovas), using either SPSS for PC (IBM, USA) or CLR anova for the Macintosh (Clear Lake Research, USA). Homogeneity of variance was tested using Mauchly's test of sphericity and, when violated, Huyn–Feldt corrections were used. When significant interactions were present, simple main effects were used to examine the effect of genotype on individual phases and blocks, using the pooled error term (in CLR anova for the Macintosh; Winer, 1971). A *P*‐value of < 0.05 was considered statistically significant throughout.

## Results

### Fear conditioning

#### Training

**Figure 2:**
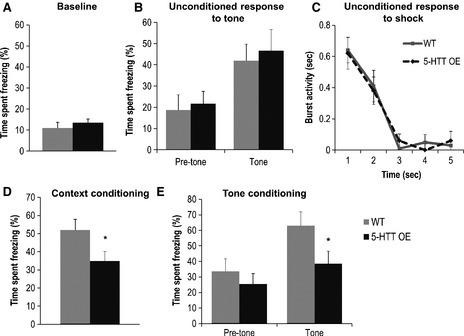
5‐HT transporter (5‐HTT) over‐expressing (5‐HTTOE) mice display impaired fear conditioning. (A) Baseline freezing during training. A 6‐min acclimatisation period occurred before the first tone–shock presentation. Mean percent time freezing (+ SEM) during this period. (B) Unconditioned response to the first tone during training. Freezing during the 4 s prior to the first tone was compared with freezing during the 4 s immediately after tone onset. Mean percent time freezing (+ SEM). (C) Unconditioned response to the first shock. The amount of rapid (‘burst’) activity was recorded for 4 s from the onset of the 0.5‐s footshock. Mean burst activity in seconds (± SEM). (D) Context fear conditioning. Graph shows the mean percent time spent freezing (+ SEM) when animals were returned to the training context 24 h later. (E) Cue fear conditioning. The 30‐s cue was re‐presented twice in a novel context and the mean percent time spent freezing (+ SEM) for time bins prior (pre‐tone) and during (tone) the tone are shown. Wild‐type mice (light bars/light solid line), *n* = 10; 5‐HTTOE mice (dark bars/dark dashed line), *n* = 10. *P < 0.05 compared with wild‐types.

During the initial 6‐min acclimatisation period, prior to the delivery of any stimuli, freezing levels were low and did not differ between the two groups [effect of genotype (*F* < 1); time bin (*F*_11,198_ = 3.42, *P* < 0.005); genotype × time bin (*F* < 1); Fig. 2A]. Analysis of the entire training session demonstrated no significant effect of genotype (*F* < 1), accompanied by a significant effect of time bin (*F*_25,450_ = 13.61, *P* < 0.001) and a significant genotype × time bin interaction (*F*_25,450_ = 1.71, *P* < 0.05). Analysis of simple main effects showed that the 5‐HTTOE mice exhibited less freezing than wild‐type mice after the second tone–shock pairing (time bins 22 and 23: *F*_1,95_ > 5.24, *P* < 0.025, data not shown).

Unconditioned responses to both the first tone and the first footshock were examined in more detail to exclude the possibility that any subsequent differences in fear conditioning were due to altered perception of either the tone or the shock. These analyses established that 5‐HTTOE mice displayed a normal unconditioned response (observed as an increase in freezing) at the onset of the first tone presentation [effect of tone (*F*_1,18_ = 16.58, *P* < 0.001); effect of genotype (*F* < 1); genotype × tone (*F* < 1); Fig. 2B], and a normal unconditioned response to the shock as shown by the increase in burst activity immediately following the first shock delivery [effect of genotype (*F* < 1); genotype × time (*F* < 1); Fig. 2C].

#### Context testing

When animals were returned to the training environment (in the absence of the auditory cue or footshock) 24 h later (Fig. 2D), 5‐HTTOE mice froze significantly less than wild‐type mice throughout the 5‐min session [effect of genotype (*F*_1,18_ = 4.76, *P* < 0.05); effect of time (*F*_9,162_ = 4.79, *P* < 0.001); genotype × time bin (*F* < 1)].

#### Cue testing

During the cue extinction test, presentation of the conditioned stimulus (CS; auditory tone) in a novel environment caused a large increase in freezing in the wild‐type mice, indicative of fear conditioning to the CS (Fig. 2E). This freezing response to the CS was attenuated in 5‐HTTOE mice. For statistical analysis, the amount of freezing observed during the 30‐s periods prior to each CS presentation was compared with that seen during the 30‐s periods of tone delivery (averaged across both tone presentations). anova revealed no overall main effect of genotype (*F*_1,18_ = 2.28, *P* > 0.10), but a main effect of CS presentation (*F*_1,18_ = 41.61, *P* < 0.0001). Importantly, there was also a significant genotype × CS presentation interaction (*F*_1,18_ = 6.15, *P* < 0.05). Analysis of simple main effects revealed that the level of freezing did not differ between genotypes during the pre‐CS period (*F* < 1, *P* > 0.20), but that the 5‐HTTOE mice displayed a reduced freezing response to the CS compared with wild‐types (*F*_1,21_ = 4.70, *P* < 0.05). Together these results clearly demonstrate a specific deficit in freezing to the shock‐associated cue in the 5‐HTTOE mice, indicating weaker fear conditioning in these animals.

Thus, 5‐HTTOE mice displayed impaired cue and context fear conditioning. Importantly, there were no group differences in baseline freezing levels prior to the delivery of any stimuli. Furthermore, there were no differences between genotypes in terms of the unconditioned responses to the first presentation of either the tone (unconditioned suppression of activity elicited by the novel tone) or the footshock (unconditioned, initial agitation induced by the shock; Bouton & Bolles, 1980). Therefore, the differences in fear conditioning do not reflect differences in the ability of the groups to perceive the tone or the shock.

### Delay/cost benefit decision‐making

**Figure 3:**
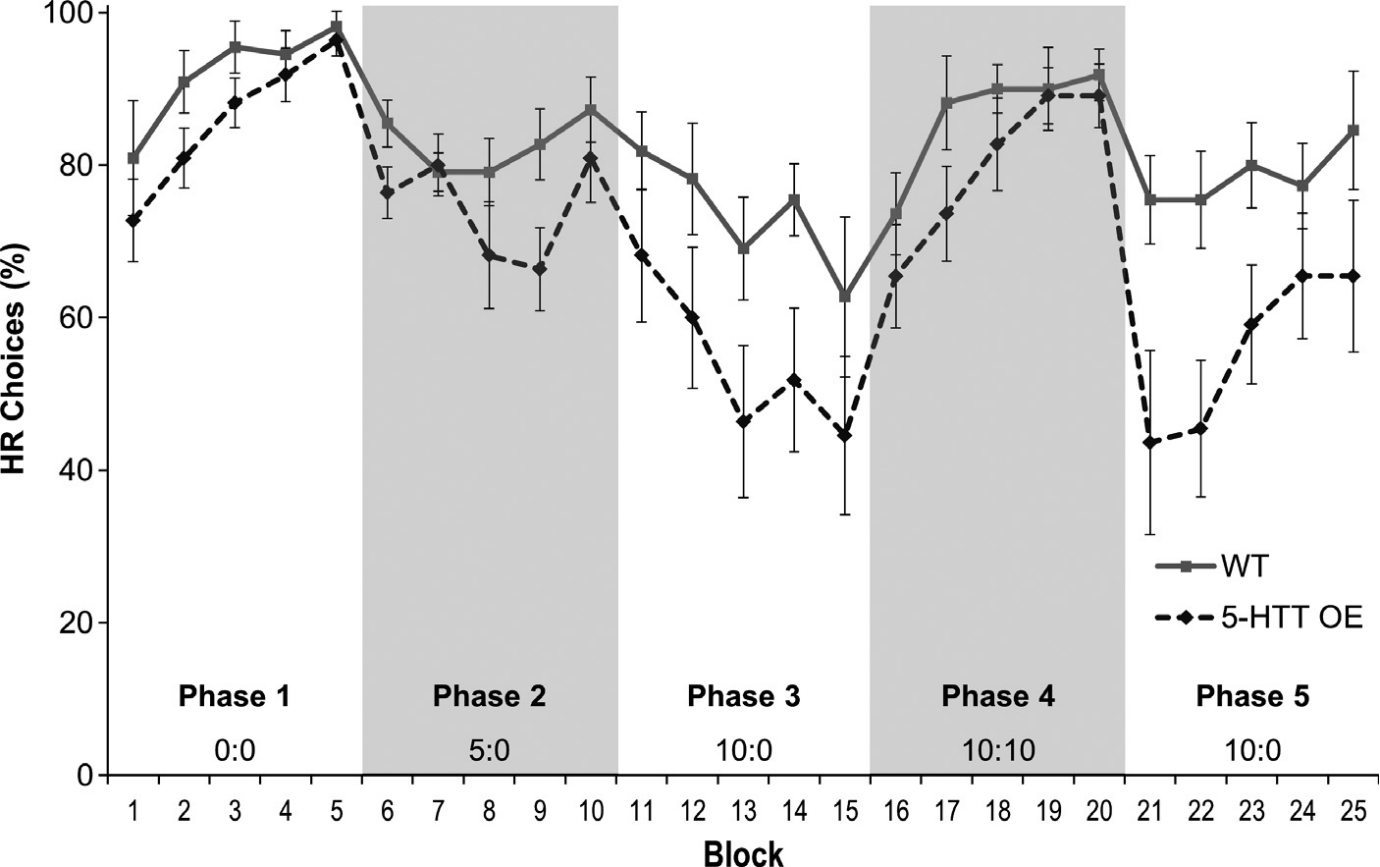
Impulsive choice on a T‐maze cost/benefit decision‐making task in 5‐HT transporter (5‐HTT) over‐expressing (5‐HTTOE) mice. Cost/benefit decision‐making experiment. Values represent the mean percent of high reward (HR) arm choices per block of 10 trials ± SEM. For Phases 1–5, the ratios for each phase depicted along the bottom of the figure represent ‘the number of seconds delay in the HR arm; number of seconds delay in the LR arm’. Twenty forced trials (10 to the HR arm and 10 to the LR arm) were given between the end of Phase 4 and the start of Phase 5. Wild‐types (light solid line; *n* = 10), 5‐HTTOE mice (dark dashed line; *n* = 11).

Inspection of Fig. 3 suggests increased impulsive choice in the 5‐HTTOE mice. During Phases 3 and 5, in which mice were required to choose between an immediate, LR and a HR that was delayed by 10 s, the 5‐HTTOE mice displayed a reduced preference for the high‐cost/high‐benefit option, compared with wild‐type controls. However, statistical analysis of the data across the whole experiment revealed a significant main effect of genotype (*F*_1,19_ = 5.00, *P* < 0.05), but, importantly, no significant genotype × phase interaction (*F*_4,76_ = 1.64, *P* = 0.19), reflecting the fact that the 5‐HTTOE mice were less likely than wild‐types to choose the HR option, irrespective of the delay conditions in the arms.

**Figure 4:**
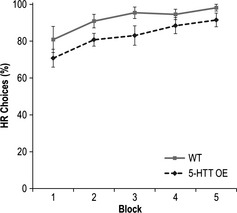
Altered reward discrimination performance in 5‐HT transporter (5‐HTT) over‐expressing (5‐HTTOE) mice. Mean percent high reward (HR) arm choices ± SEM during Phase 1 of the decision‐making experiment (acquisition of the reward magnitude discrimination), showing data for all animals that completed this phase. Wild‐types (light solid line; *n* = 11), 5‐HTTOE mice (dark dashed line; *n* = 13).

This could reflect a reduced sensitivity to reward in the 5‐HTTOE mice. Indeed, although the animals included in the final analysis were well matched for performance by the end of the initial reward magnitude discrimination training (Fig. 3; Phase 1), with both groups choosing the HR option on more than 90% of trials (main effect of genotype for Phase 1: *F*_1,19_ = 1.64, *P* > 0.20), it is important to note that two transgenic animals (out of 13) were excluded at the end of Phase 1 for failing to attain the performance criterion of > 80% HR choices, and so are not included in this analysis. One wild‐type mouse was injured and was also excluded at this point. Notably, if these three animals were included in a re‐analysis of Phase 1, then there was a significant main effect of genotype (*F*_1,22_ = 4.35, *P* < 0.05; Fig. 4), suggesting that 5‐HTTOE mice were in fact less able to acquire the original differential reward discrimination than wild‐types [effect of block (*F*_4,88_ = 9.37, *P* < 0.001); genotype × block (*F* < 1)].

Nevertheless, to confirm that over‐expression of the 5‐HTT affected the acquisition of the reward magnitude discrimination, a further cohort of experimentally naïve, wild‐type and 5‐HTTOE mice were trained as before. Again, there was a significant impairment in the 5‐HTTOE mice, reflecting the fact that they were less likely to choose the HR option during training on the reward discrimination, even in the absence of any delays [main effect of genotype (*F*_1,31_ = 8.54, *P* < 0.01), data not shown].

### Spatial memory

To assess whether this impairment in the 5‐HTTOE mice on the reward magnitude discrimination task reflected a general problem with spatial memory performance, mice were compared on a battery of standard spatial memory tests.

#### Rewarded alternation

**Figure 5:**
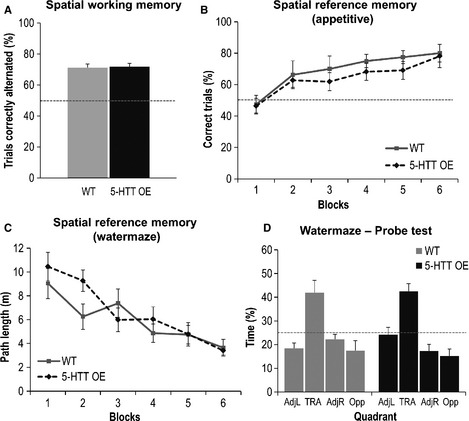
5‐HT transporter (5‐HTT) over‐expressing (5‐HTTOE) mice display normal spatial learning and memory. (A) Spatial working memory (non‐matching to place) rewarded alternation task. The graph shows the mean percent correct alternations (+ SEM) from 40 trials. The dotted line indicates chance performance at 50% correct choices. Wild‐type (light bar; *n* = 10), 5‐HTTOE mice (dark bar; *n* = 11). (B) Appetitively motivated spatial reference memory Y‐maze task. The graph shows mean percent correct choices (± SEM) per block of 10 trials. Wild‐type (light solid line; *n* = 8), 5‐HTTOE mice (dark dashed line; *n* = 11). Horizontal dotted line indicates chance performance at 50% correct choices per block. (C) Morris water‐maze. The graph shows the mean distance swum [pathlength (m) ± SEM] to reach the hidden platform over six blocks of trials (four trials per block). Wild‐type (light solid line; *n* = 10), 5‐HTTOE mice (dark dashed line; *n* = 11). (D) Water‐maze probe test. Twenty‐four hours after block 6 of training, animals were returned to the pool with the platform removed and the amount of time spent swimming in each quadrant was recorded. Quadrant abbreviations: AdjL, adjacent left; TRA, training; AdjR, adjacent right; Opp, opposite. Mean percent time in quadrant (+ SEM) is displayed. The dotted line indicates chance performance at 25% time spent in a given quadrant. Wild‐type (light bars; *n* = 10), 5‐HTTOE mice (dark bars; *n* = 11).

Both groups of mice showed a good level of spatial working memory (alternation) performance on the T‐maze (Fig. 5A). 5‐HTTOE mice did not differ significantly from wild‐type mice on the percentage of trials correct (*F* < 1). Thus, short‐term spatial memory was preserved in the 5‐HTTOE mice.

#### Reference memory Y‐maze

Both groups of mice acquired the appetitively motivated spatial reference memory Y‐maze task, and learned to choose correctly the target arm that contained the milk reward, as defined by its allocentric spatial location. Performance of the 5‐HTTOE mice did not differ from that of wild‐type mice [genotype (*F* < 1); block (*F*_5,85_ = 10.70, *P* < 0.001); genotype × block (*F* < 1); Fig. 5B].

#### Morris water‐maze

Similarly, compared with wild‐type controls, 5‐HTTOE mice were not impaired on the standard, fixed location, hidden escape platform version of the Morris water‐maze task. Mice in both groups learned the spatial location of the hidden platform. Analysis of the pathlength data confirmed that mice became more efficient at finding the platform as training continued [main effect of block (*F*_5,95_ = 14.94, *P* < 0.001)], and that spatial learning in 5‐HTTOE mice did not differ significantly from that of wild‐types [genotype (*F* < 1); genotype × block (*F*_5,95_ = 1.75, *P* = 0.14; Fig. 5C]. A similar pattern of results was obtained when the time taken for the animals to reach the platform was analysed (data not shown).

A probe test was then performed 24 h after the final block of training (Fig. 5D). Both groups of mice spent the largest amount of time searching in the quadrant of the pool that had formerly contained the escape platform. Analysis of the probe test data found a significant effect of quadrant, but no genotype × quadrant interaction [effect of quadrant (*F*_2,57_ = 17.87, *P* < 0.01) genotype by quadrant interaction (*F* < 1)]. Note that for analysis of the time spent in each quadrant during the probe tests, *P*‐values were adjusted to reflect a reduction in the degrees of freedom in both the main effect of quadrant and the group by quadrant interaction (because the fourth quadrant data point was never independent of the other three quadrants). A further comparison of the time spent in the training quadrant also failed to reveal a group difference [effect of genotype (*F* < 1)]. In addition, the 5‐HTTOE mice did not differ from wild‐types in terms of the number of target annulus crossings (*F* < 1; data not shown), confirming that the two groups had learned about the location of the platform to an equal degree.

### Neurochemistry

**Figure 6:**
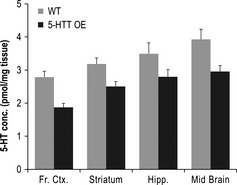
Reduced 5‐HT levels across a number of brain regions in 5‐HT transporter (5‐HTT) over‐expressing (5‐HTTOE) mice. Mean regional tissue 5‐HT concentration (+ SEM) for wild‐types (light bars; *n* = 8) and 5‐HTTOE mice (dark bars; *n* = 10).

5‐HTTOE mice displayed significantly lower overall levels of brain tissue 5‐HT compared with wild‐types (*F*_1,16_ = 19.35, *P* < 0.001; Fig. 6). There was no genotype × region interaction (*F*_4,64_ = 1.48, *P* = 0.24), indicating that the reduction in 5‐HT was similar across all the brain regions assayed.

## Discussion

Genetically modified mice over‐expressing the 5‐HTT exhibited impairments on selected appetitive and aversive learning and memory tasks relative to their wild‐type controls. 5‐HTTOE mice displayed impaired fear conditioning, both to a punctate auditory cue paired with a mild footshock, and to the environmental context in which the shock was received. These mice also displayed impulsive choice on an appetitively motivated, cost/benefit decision‐making T‐maze task. Further inspection of the data suggested that the 5‐HTTOE mice had a reduced preference for the HR option, irrespective of any delay, and were also less inclined to choose the goal arm associated with the HR arm during reward discrimination training when there was no delay to reinforcement in either arm. This suggests a deficit in the processing of reward magnitude in these mice. Notably, this deficit appears selective in that the 5‐HTTOE mice displayed normal acquisition and performance on appetitively motivated, spatial working and reference memory tasks in which mice were required to choose between locations associated with either reward or no reward. Furthermore, the mice displayed normal performance on the standard, fixed location, hidden escape platform version of the open‐field, Morris water‐maze task.

Thus, our data show that relative to wild‐type controls, 5‐HTTOE mice display reduced sensitivity to both positive and negative reinforcement on associative learning tasks, which may be relevant to the development of abnormal emotional phenotypes. These mice have increased 5‐HTT levels within the physiological range, which result in reduced whole tissue levels of 5‐HT across a number of brain regions that have been sampled here and elsewhere (Jennings *et al*., 2006, 2008; Barkus *et al*., 2014). Therefore, these data are consistent with recent findings from human gene association studies suggesting that although *l* homozygosity in the 5‐HTTLPR may be protective for anxiety disorders, it may itself be maladaptive, and a potential risk factor for certain neuropsychiatric conditions (Hu *et al*., 2006; Wendland *et al*., 2008; Glenn, 2011). This is also potentially consistent with recent suggestions that low‐activity serotonin transporter genotypes are also associated with both ‘for‐better‐and‐for‐worse’ phenotypes (Homberg & Lesch, 2011; Homberg & van den Hove, 2012).

### 5‐HTT over‐expression and reduced whole tissue serotonin

The observation that 5‐HTT over‐expression leads to reduced whole tissue levels of brain 5‐HT confirms previous findings (Jennings *et al*., 2006, 2008; Barkus *et al*., 2014). This result is unexpected in that 5‐HTT KO mice and rats also have this neurochemical phenotype (Kim *et al*., 2005; Homberg *et al*., 2007), and in simple terms increased 5‐HT uptake might be expected to increase tissue 5‐HT. Although 5‐HTTOE mice demonstrate the predicted reduction in extracellular 5‐HT levels (as measured with both microdialysis and fast‐scan cyclic voltammetry; Jennings *et al*., 2006, 2010), markers of 5‐HT synthesis and metabolism are unchanged (Jennings *et al*., 2006). One possibility is that the 5‐HTTOE mice may have a 5‐HT storage deficit, given that the vesicular storage compartment is likely to be a major determinant of the whole tissue neurotransmitter levels (Jennings *et al*., 2006). This could reflect abnormal interactions between the 5‐HTT and proteins involved in vesicle formation and utilization. It is not inconceivable that an abnormal interaction between the transporter and these vesicular proteins could generate reduced whole tissue levels of 5‐HT in both the over‐expressing and KO mice, although this is speculative and requires further research.

### 5‐HTT expression levels and emotionality

There has been much research into associations between 5‐HTTLPR polymorphisms, 5‐HTT expression levels, and behavioural and emotional phenotypes (Homberg & Lesch, 2011; Murphy & Moya, 2011). Interpretation of these findings is complicated by failed replications (Risch *et al*., 2009; Uher & McGuffin, 2010), and evidence for a small effect of individual polymorphisms on the phenotypic expression of complex traits (Flint & Munafo, 2007). However, genetically modified animals with altered 5‐HTT expression levels demonstrate striking phenotypic changes in emotionality (Murphy & Lesch, 2008). For example, 5‐HTTOE mice exhibit reduced anxiety across a range of ethological, unconditioned tests, whereas 5‐HTT KO mice display increased anxiety (Holmes *et al*., 2003; Jennings *et al*., 2006; Line *et al*., 2011). Similarly, in the present study, 5‐HTTOE mice exhibited reduced fear (in terms of freezing levels) to cues that have been previously paired with footshock (see also Barkus *et al*., 2014), whereas 5‐HTT KO mice and rats are inclined to show increased levels of conditioned freezing to fear‐inducing cues [although in different studies these increases in freezing have been attributed to either altered fear extinction (Nonkes *et al*., 2012; Shan *et al*., 2014), or altered fear extinction recall (Wellman *et al*., 2007)]. Furthermore, naturally occurring variation in 5‐HTT levels in humans (whether genetically driven or not), has been shown to impact upon the processing of emotionally relevant stimuli, with evidence for reduced amygdala activation in humans with higher 5‐HTT expression levels (Rhodes *et al*., 2007). Importantly, the present data add to these observations by clearly showing that mice that model physiologically relevant increases in 5‐HTT expression levels in humans display robust deficits in behaviours (such as fear conditioning), which are believed to be mediated by the amygdala.

### Fear conditioning is impaired in 5‐HTTOE mice

The deficit in fear conditioning, observed in both male and female 5‐HTTOE mice (see also Barkus *et al*., 2014), is consistent with the observation that fear conditioning is reduced in human subjects with the *l* allele 5‐HTTLPR polymorphism compared with subjects with the *s* allele, as measured using skin conductance responses following the pairing of simple visual stimuli with electric shock (Garpenstrand *et al*., 2001; Lonsdorf *et al*., 2009). These observations are also potentially consistent with findings of reduced amygdala activation in response to emotional stimuli such as fearful faces, in *l* allele human subjects (Hariri *et al*., 2002), and in subjects with reduced 5‐HTT availability as measured using positron emission tomography (Rhodes *et al*., 2007). Indeed, we recently demonstrated reduced amygdala activity in 5‐HTTOE mice in response to an aversive CS during a fear conditioning task, using a haemodynamic measure analogous to the functional magnetic resonance imaging blood oxygen level‐dependent signal (Barkus *et al*., 2014). The importance of the amygdala for fear conditioning is well established (Davis, 2004; Phelps & LeDoux, 2005), both from human imaging studies (Buchel *et al*., 1998; LaBar *et al*., 1998; Hasler *et al*., 2007) and from lesion studies in animals (Phillips & LeDoux, 1992; Campeau & Davis, 1995). Amygdala lesions have been reported to disrupt conditioned freezing, both to a tone CS and to contextual cues (Phillips & LeDoux, 1992). Thus, deficits in fear conditioning in 5‐HTTOE mice are consistent with the possibility of reduced amygdala activity in these animals, paralleling the findings in human subjects with the *l* allele 5‐HTTLPR polymorphism (Rhodes *et al*., 2007), although it is important to note that altered 5‐HT neurotransmission in other brain structures may also contribute to this phenotype. It is also worth pointing out that with extended training 5‐HTTOE mice can successfully learn to associate auditory cues with aversive stimuli [e.g. during a discriminative fear conditioning paradigm in which the mice had to discriminate between a CS+ cue that was associated with shock and a neutral CS− cue (Barkus *et al*., 2014)], suggesting a subtle effect on the rate of learning.

It is also worth noting that the unconditioned response to the first footshock received, which is a rapid burst of activity, was well matched across the two genotypes (Fig. 2C). Thus, attempted undirected escape responses appeared undiminished in 5‐HTTOE mice, in contrast to impairments in fear conditioning‐induced freezing. This is consistent with the idea that alterations in the 5‐HT system may differentially affect different responses to threatening stimuli within a hierarchical defence system (Deakin & Graeff, 1991).

### Altered reward processing in 5‐HTTOE mice

Behavioural deficits in the 5‐HTTOE mice were not limited to aversive learning paradigms. These mice also displayed abnormal behaviour on an appetitively motivated, cost/benefit decision‐making T‐maze task. 5‐HTTOE mice displayed a reduced preference for the goal arm associated with the HR, compared with wild‐type controls. That this deficit was particularly pronounced during testing when there was a 10‐s delay to reinforcement in the HR goal arm suggests impulsive choice behaviour in these animals. This finding, together with the observations here (Fig. 6) and elsewhere that 5‐HTTOE mice have reduced tissue 5‐HT content and release (Jennings *et al*., 2006, 2010; Barkus *et al*., 2014), is consistent with previous studies showing a similar change in impulsivity in response to pharmacological 5‐HT depletion (Bizot *et al*., 1999; Mobini *et al*., 2000; Denk *et al*., 2005; but see also Isles *et al*., 2005 for mice with reduced 5‐HT but no change in impulsivity). This phenotype in the 5‐HTTOE mice connects with an emerging literature of an association between rare over‐expressing 5‐HTT gene variants and obsessive compulsive disorders as well as impulsive traits, which have long been linked to 5‐HT deficits (Hu *et al*., 2006; Wendland *et al*., 2008; Glenn, 2011; Voyiaziakis *et al*., 2011).

Notably, however, the reduced choice of the HR arm was not limited to the delay conditions. 5‐HTTOE mice also displayed reduced HR choices during the initial acquisition of the reward magnitude discrimination, in the absence of any delays. This suggests a deficit in reward processing or insensitivity to reward magnitude. It is worth pointing out that our original intention was to investigate whether the 5‐HTTOE mice would behave differently during tests of cost/benefit decision‐making, given the important role that serotonin plays in these kinds of tasks (Bizot *et al*., 1999; Mobini *et al*., 2000; Denk *et al*., 2005; see Homberg, 2012 for review). *A priori*, we did not necessarily predict that there would be an effect on the acquisition of the task (i.e. learning the reward discrimination prior to the introduction of delays). Instead, we were predicting that the deficit might only appear once the animals had to integrate information about rewards and delays. However, this was not the case. There was a deficit in the 5‐HTTOE mice irrespective of any delays.

Importantly, we have shown previously that over‐expression of the 5‐HTT does not affect feeding behaviours or satiety in food‐deprived mice (Pringle *et al*., 2008). Any impairment in reward processing is likely to be subtle, or limited to a specific aspect of reward information processing, as 5‐HTTOE mice appeared normal on tests of spatial working and reference memory in which they were required to choose between options that were associated with either reward or no reward. These different outcomes across the studies could simply reflect the difficulty or sensitivity of the various tasks, with absolute ‘all vs. none’ discriminations being easier to solve than relative reward discriminations. In this respect it is worth noting, however, that the actual magnitude of the difference during reward discrimination training on the decision‐making task (0.2 mL condensed milk) is greater than the magnitude of the difference in the spatial working and reference memory tasks (0.1 mL). Nevertheless, the fact that during the initial reward magnitude discrimination training for the decision‐making task, both response options were associated with reward (and hence the animals’ response choices were ambiguous), may make this task more difficult than acquisition of the spatial reference memory Y‐maze task.

### Reduced sensitivity to positive and negative reinforcers

Thus, mice over‐expressing the 5‐HTT exhibited impairments on certain appetitive and aversively motivated learning tasks, although this was by no means a global cognitive impairment. Specifically, there were impairments on the aversive fear conditioning task and the appetitive reward magnitude discrimination learning paradigm (i.e. ‘prior’ to the introduction of any delays). Both the fear conditioning reward magnitude discrimination learning tasks involve the formation of associations between stimuli and outcomes. The 5‐HTTOE mice demonstrate impairments on both tasks, suggesting that there may be a general (albeit subtle) problem with associative learning for both aversive and appetitive outcomes. These results are consistent with a role for 5‐HT in the processing of both aversive and rewarding stimuli (McCabe *et al*., 2010). These learning deficits were observed in several different behavioural tasks that involved a variety of different cue types. 5‐HTTOE mice were impaired in both cue (tone) and context fear conditioning, and in the appetitive T‐maze task that could be solved using allocentric and/or egocentric spatial cues. Thus, in the context of the present study, the deficits appeared to be independent of the type of cue.

The possibility of altered reward processing in 5‐HTTOE mice is consistent with numerous studies in humans and animals that suggest a role for 5‐HT in reward systems, particularly in terms of reward sensitivity (Rogers *et al*., 2003; Bari *et al*., 2010). Deficits in the aversive, fear‐conditioning paradigm suggest that the impairments in the 5‐HTTOE mice are not limited to reward systems but extend to the processing of negative reinforcement. Indeed, the latter is also consistent with a large literature implicating 5‐HT and, more specifically, the 5‐HTT in fear learning (Garpenstrand *et al*., 2001; Hariri *et al*., 2002; Rhodes *et al*., 2007). The current data are also broadly consistent with studies showing that gene polymorphisms in the 5‐HTTLPR influence the outcome when individuals are required to choose between options that are associated with different levels of reward and/or punishment (Finger *et al*., 2007; Blair *et al*., 2008).

## Conclusions

To conclude, the present data further support a key role for variation in 5‐HTT expression levels in emotional learning. Importantly, the behavioural impairments observed in the 5‐HTTOE mice suggest that not only reduced, but also increased, expression of the 5‐HTT is detrimental to emotional processing. Indeed, these data are consistent with the emerging viewpoint that the *l/l* 5‐HTT genotype may also be a risk factor for the development of certain neuropsychiatric conditions including obsessive compulsive behaviours, and for psychopathic traits characterised by emotional insensitivity.
